# Does meniscectomy have any advantage over conservative treatment in middle-aged patients with degenerative medial meniscus posterior root tear?

**DOI:** 10.1186/s12891-021-04632-8

**Published:** 2021-08-28

**Authors:** Nam-Hun Lee, Hyoung-Yeon Seo, Myung-Jin Sung, Bo-Ram Na, Eun-Kyoo Song, Jong-Keun Seon

**Affiliations:** 1 Department of Orthopaedic Surgery, Chonnam National University Hwasun Hospital and Medical School, 322 Seoyang-ro, Hwasun-gun, Chonnam 58218 Republic of Korea; 2 Department of Orthopaedic Surgery, Chonnam National University Hospital, 42 Jebongro, Donggu, Gwangju, 61469 Republic of Korea

**Keywords:** Medial meniscus posterior root tear, Arthroscopic meniscectomy, Conservative treatment, Osteoarthritis

## Abstract

**Background:**

The best treatment for degenerative medial meniscus posterior root tear (MMPRT) remains controversial. This study aimed to compare the clinical and radiological outcomes of arthroscopic meniscectomy and conservative treatment for degenerative MMPRT.

**Methods:**

From January 2007 to December 2014, 146 patients (Meniscectomy group, 90; Conservative group, 56) were evaluated. Clinical outcomes were assessed using the Visual Analog Scale, International Knee Documentation Committee subjective scoring scale, Tegner activity scale, and Lysholm knee scoring scale at the final follow-up. Radiologic outcomes evaluated the progression of osteoarthritis (OA) according to the Kellgren-Lawrence (K-L) classification. We compared the hip-knee-ankle angle (HKAA), medial proximal tibial angle, tibial posterior slope angle, and width of medial joint space. After an average follow-up of 6.3 years, the survivorship was analyzed using the Kaplan–Meier method.

**Results:**

All clinical outcomes were significantly improved in both groups after treatment, with no significant differences between the two groups at the final follow-up. The progression of OA according to the K-L classification, HKAA and width of medial joint space was significantly advanced in the meniscectomy group (*p* = 0.03, 0.04, 0.03, respectively). The 10-year survival rates in the meniscectomy and conservative groups were 87 and 88%, respectively.

**Conclusions:**

This study demonstrated that both conservative treatment and meniscectomy provided symptomatic relief. However, it was confirmed that OA progression was more severe in the meniscectomy. We conclude that arthroscopic meniscectomy had no advantage over conservative treatment in terms of clinical outcomes and OA progression in middle-aged patients with MMPRT.

**Level of evidence:**

Level III; retrospective comparative study.

## Background

Meniscus roots are a vital component of the meniscus as they anchor the meniscus to the tibial plateau and disperse axial loads into hoop stresses during loading. Medial meniscus posterior root tear (MMPRT) was defined as a radial tear within 9 mm of the posterior bony attachment of the medial meniscus or posterior root avulsion. MMPRT leads to the loss of hoop tension and load transmissibility in the meniscus, which results in a biomechanical condition similar to that observed after total meniscectomy [1]. A high incidence (27.8%) of MMPRT has been reported in Asians because of lifestyles, including frequent squatting and sitting on the floor with legs folded [2]. MMPRT tends to have a worse prognosis because it commonly occurs in patients aged over 50 years, whose meniscal tissue may have degenerated and who may have low healing potential [2, 3].

Treatment options for MMPRT include conservative treatment, meniscectomy, and root repair. Historically, patients with MMPRT have been treated with conservative treatment or a partial meniscectomy [4]. In recent years, there has been increasing interest in root repair because meniscectomy has been reported to increase the risk of osteoarthritis (OA) [5]. It is well known that acute traumatic MMPRT without OA should be repaired whenever possible to restore meniscal hoop tension and to prevent early arthritic progression [6–9]; however, a large proportion of meniscal root tears seen in clinical practice involve degenerative MMPRTs in middle-aged or older patients [10–12]. Hence, surgical repair is not always feasible in the population at risk of these tears [10, 13] due to substantial degeneration of the meniscal tissue and concurrent OA [2, 3, 14]. Therefore, the best treatment for degenerative MMPRT remains controversial [2, 8, 15].

Although the short-term clinical results of MMPRT repair have been encouraging [8, 16, 17], meniscectomy for MMPRT has been traditionally used because it is relatively easier than the repair, and symptomatic improvement can be expected by removing the source of mechanical pain [2, 3, 5]. Even though the results were heterogeneous, recent studies have reported that conservative treatment and meniscectomy can be a good option for selected patients with good prognostic factors [18–21], In addition, conservative treatment with exercise therapy has also been reported to be a reasonable treatment option for middle-aged patients with early OA [22, 23]. Therefore, we decided to investigate the failure rate, clinical results, and OA progression after arthroscopic meniscectomy and conservative treatment for degenerative MMPRT in middle-aged patients.

## Methods

After obtaining permission from the institutional ethical committee, we retrospectively reviewed our database of patients diagnosed with meniscus root tear from January 2007 to December 2014. MMPRT was defined as a radial tear within 9 mm of the posterior bony attachment of the medial meniscus or posterior root avulsion was diagnosed on MRI by the absence of an identifiable meniscus or high signal replacing the normal dark meniscal signal (“ghost sign”) in the sagittal plane, a vertical linear defect at the root in the coronal plane, and a radial linear defect at the posterior insertion in the axial plane [24]. Initial OA grade and OA progression in the medial compartment at the last follow-up were graded according to the Kellgren-Lawrence (K-L) classification system [25].. The K-L classification was originally described using AP knee radiographs. Each radiograph was assigned a grade from 0 to 4, which they correlated to increasing severity of OA, with Grade 0 signifying no presence of OA and Grade 4 signifying severe OA.

**Fig. 1:**
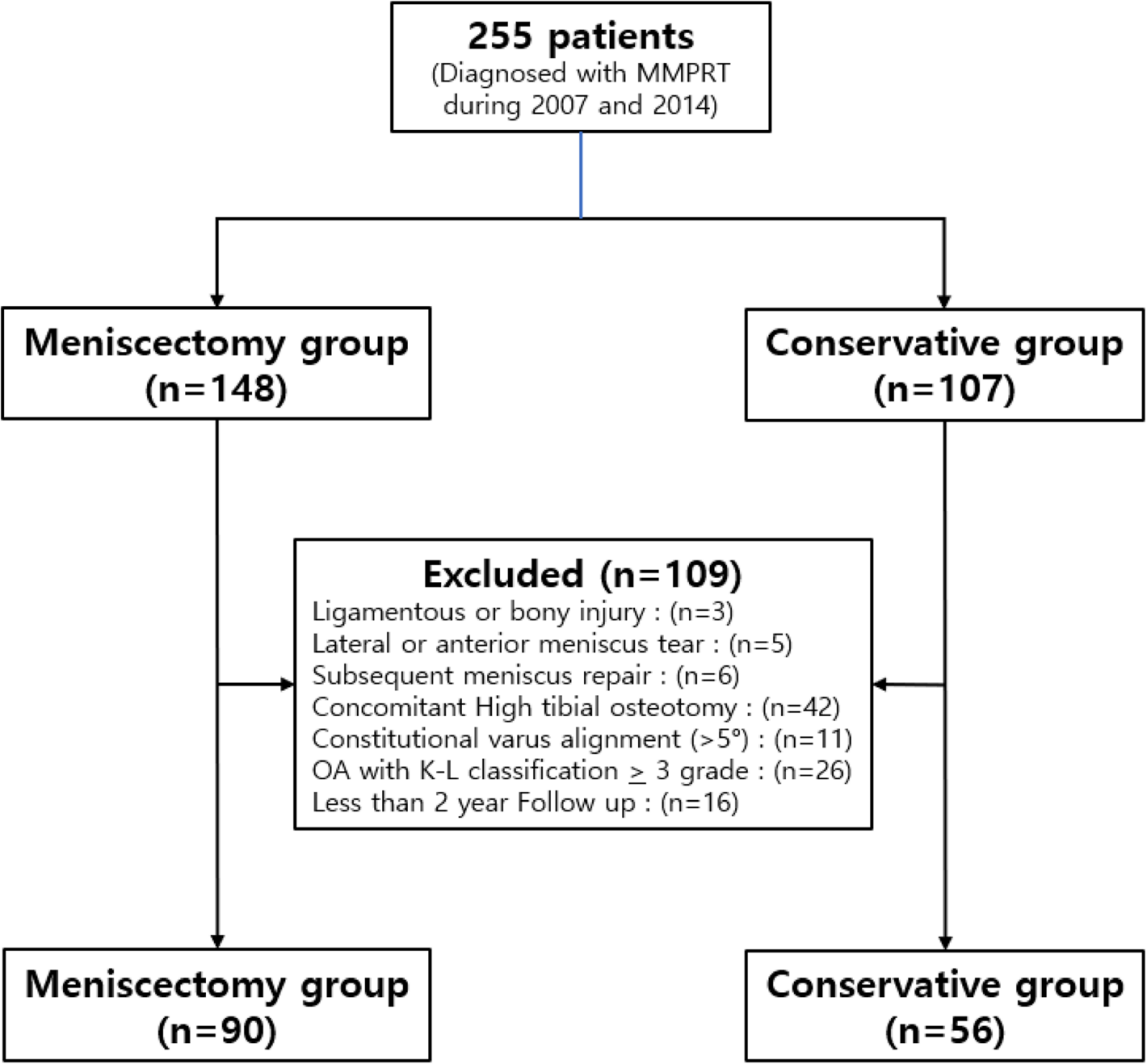
Patient selection flowchart. MMPRT, Medial meniscus posterior root tear; K-L, Kellgren-Lawrence

The inclusion criteria were as follows: (1) diagnosis of complete medial meniscus posterior root avulsion or complete radial tear adjacent (within 9 mm) to the medial meniscus posterior root by a musculoskeletal radiologist [24], (2) presentation of clinical symptoms that were correlated with MRI findings, and (3) arthroscopic complete or partial meniscectomy or conservative treatment. The exclusion criteria were as follows: (1) previous or subsequent ligamentous knee injury, such as a high-energy traumatic injury to the root attachment, (2) concomitant traumatic tibial plateau fracture, (3) associated with lateral or anterior meniscus tears, (4) subsequent meniscal repair after diagnosis, (5) concomitant high tibial osteotomy caused by varus malalignment, (6) constitutional varus alignment > 5°, (7) presence of grade > III OA based on the K-L classification and severe chondral defect/injuries, and (8) less than 2 years of clinical follow-up. Out of 255 patients, 146 (meniscectomy group, *n* = 90; conservative treatment group, *n* = 56) were included in the study. (Fig. 1).

Conservative treatment included daily nonsteroidal anti-inflammatory drugs for 4–8 weeks and supervised physical therapy twice a week, including activity modification for at least 6 weeks. The goal of physical therapy was to reduce pain, restore full range of motion, and improve knee function. Physical therapy consisted of exercises for muscle strength and endurance. Each patient visited a physiotherapy office and followed a standard exercise program twice a week.

The indication for arthroscopic meniscectomy was MMPRT in patients with persistent knee pain with mechanical symptoms affecting activities of daily living, despite conservative treatment for 3 months after MMPRT was diagnosed. The treatment modality was chosen by the patients after discussion with surgeon based subjective symptoms.

The first visit and follow-up clinical findings were assessed using the Visual Analog Scale (VAS), International Knee Documentation Committee (IKDC) subjective scoring scale, Tegner activity scale, and Lysholm knee scoring scale. If the patients underwent conversion to total knee arthroplasty (TKA), unicompartmental knee arthroplasty (UKA), or high tibial osteotomy (HTO), the final clinical outcomes were assessed just before the conversion.

Regarding radiological outcomes, we compared the hip-knee-ankle angle (HKAA), medial proximal tibial angle (MPTA), tibial posterior slope angle (TPSA), and width of medial joint space between the groups. We also evaluated the progression of OA in the medial compartment of the knee according to the K-L classification system. To determine the intra- and inter-observer reliabilities of the radiographic outcomes, two investigators performed all the radiographic assessments twice (1-week intervals). Intraclass correlation coefficients were used for inter- and intra-observer reliability assessments. After an average follow-up of 6.3 years, the survivorship was analyzed using the Kaplan–Meier method. The endpoint of survival was conversion to TKA or UKA or HTO in the same knee.

### Statistical analysis

Statistical analysis was performed using SPSS version 20.0 (SPSS, Chicago, IL). A *P*-value < 0.05 was considered statistically significant. Pearson’s chi-square test and Fisher’s exact test were used to determine the statistical significance of differences in categorical variables. For continuous variables, independent t-test was used for normally distributed variables and the Mann-Whitney U test was used to compare non-normally distributed variables between groups. The Wilcoxon signed-rank test was used to compare the preoperative and last follow-up clinical outcomes in each group. Kaplan–Meier survival analysis was performed to evaluate the time-dependent rate of conversion to arthroplasty, and a hazard ratio was created via a Cox proportional hazards model.

## Results

The demographic data were similar in both groups. With no significant differences between the two groups (Table 1). All inter- and intra-observer intraclass correlation coefficients showed good agreement with the reliability of radiographic measurement (> 0.85). There were no significant differences between the meniscectomy and conservative groups in terms of preoperative HKAA (*p* = 0.76), MPTA (*p* = 0.23), TPSA (*p* = 0.73) or width of medial joint space (*p* = 0.19) (Table 2).

**Table 1:** Comparison of the demographics

	Meniscectomy group (*n* = 90)	Conservative group (*n* = 56)	*P*-value
Sex^a^ (M/F)	31 / 59	17 / 39	0.61
Age^b^ (y)	55.5 ± 8.6	57.7 ± 8.1	0.13
BMI^b^ (kg/m^2^)	25.5 ± 3.8	25.4 ± 2.0	0.84
Follow-up duration^b^ (y)	6.4 ± 3.7	6.1 ± 4.0	0.60

^a^Pearson’s chi-square test, ^b^Independent t-test. Data are presented median ± standard deviation. The *P*-values reflect the results of inter-group comparisons, with *p* < 0.05 indicating significance.*BMI* Body mass index;

**Table 2:** Comparison of the preoperative variables

	Meniscectomy group (*n* = 90)	Conservative group (*n* = 56)	*P*-value
HKAA^b^ (varus, °)	2.9 ± 2.5	2.7 ± 2.4	0.76
MPTA^b^ (°)	87.3 ± 3.0	86.6 ± 3.3	0.23
TPSA^b^ (°)	7.0 ± 4.4	6.8 ± 3.5	0.73
K-L grade^a^ (I/II) Width of medial joint space‡ (mm)	3/87 4.7 ± 1.2	5/51 4.6 ± 1.1	0.26 0.19

^a^Pearson’s chi-square test, ^b^Independent t-test. Data are presented median ± standard deviation. The *P*-values reflect the results of inter-group comparisons, with p < 0.05 indicating significance.*HKAA* Hip-knee-ankle angle, *MPTA* Medial proximal tibial angle, *TPSA* Tibial posterior slope angle, *K-L* Kellgren-Lawrence

On comparing the two groups, the VAS score (*p* = 0.07), IKDC subjective score (*p* = 0.18), Tegner activity scale score (*p* = 0.08), and Lysholm knee score (*p* = 0.53) showed no significant differences between the two groups at the final follow-up. Because baseline clinical outcomes were statistically different, the degree of improvement were also compared, and IKDC subjective score (*p* = 0.19), Tegner activity scale score (*p* = 0.67), and Lysholm knee score (*p* = 0.36) showed no significant differences between the two groups. (Table 3).

**Table 3:** Comparison of the clinical outcomes

	Meniscectomy group (n = 90)	Conservative group (*n* = 56)	*P*-value†
VAS			
First visit	5.9 ± 0.8	4.3 ± 1.3	0.00
Last follow-up	4.3 ± 1.5	3.8 ± 1.2	0.07
Improvement	1.7 ± 1.3	0.8 ± 1.2	0.00
*P* value‡	0.00	0.01	
IKDC subjective scoring scale			
First visit	26.3 ± 8.3	30.6 ± 9.8	0.00
Last follow-up	33.9 ± 9.3	38.1 ± 8.8	0.18
Improvement	8.6 ± 8.9	8.9 ± 9.3	0.19
P value‡	0.00	0.01	
Tegner activity scale			
First visit	2.3 ± 0.9	2.7 ± 0.9	0.03
Last follow-up	2.8 ± 1.1	3.1 ± 0.9	0.08
Improvement	0.6 ± 1.0	0.5 ± 0.8	0.67
*P* value‡	0.00	0.03	
Lysholm knee scoring scale			
First visit	50.9 ± 8.7	54.1 ± 8.9	0.00
Last follow-up	65.5 ± 9.4	67.0 ± 10.8	0.53
Improvement	14.9 ± 9.1	12.5 ± 9.8	0.36
*P* value‡	0.00	0.00	

†Mann-Whitney U test for analysis of difference. ‡Wilcoxon signed-rank test for comparison of clinical outcomes between preoperative and last follow up points. *Values are presented as means and standard deviations. The *p*-values reflect the results of inter-group comparisons, with *p* < 0.05 indicating significance

The progression of varus in HKAA was significantly advanced in the meniscectomy group (*p* = 0.04). In addition, the width of medial joint space at the last follow up was less in the meniscectomy group(*p* = 0.03), and OA progression according to the K-L classification was found to have advanced in the meniscectomy group (*p* = 0.03). Progression to grade III from grade II was observed in 45 cases and to grade IV from grade II was observed in 17 cases in the meniscectomy group. Progression to grade III from grade II was observed in 26 cases and to grade IV from grade II was observed in three cases in the conservative group. Grade I remained as grade I in both groups. (Table 4).

**Table 4:** Comparison of the radiological outcomes

	Meniscectomy group (n = 90)	Conservative group (n = 56)	*P-*value†
HKAA† (varus, °)	4.3 ± 2.3	3.6 ± 2.5	0.04
Width of medial joint space† (mm)	3.1 ± 1.1	3.5 ± 1.2	0.03
K-L grade‡			
Grade I	3	5	0.03
Grade II	25	22	
Grade III	45	26	
Grade IV	17	3	

†Independent t-test. ‡Fisher’s exact test. The *p-*values reflect the results of inter-group comparisons, with p < 0.05 indicating significance. *HKAA* Hip-knee-ankle angle, *K-L* Kellgren-Lawrence

**Fig. 2:**
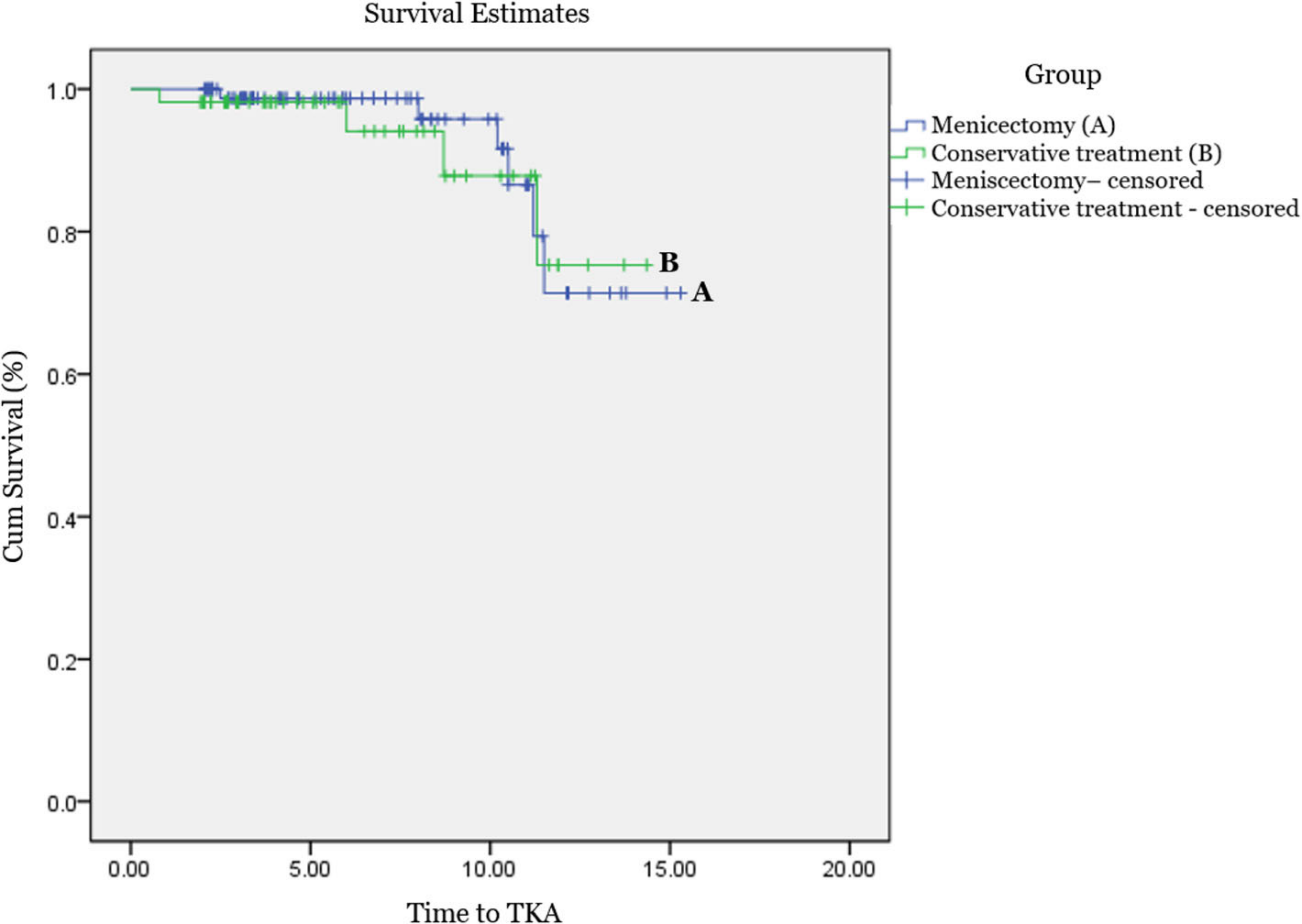
Kaplan-Meier analysis of joint survival after meniscectomy and conservative treatment

In terms of the survivorship analysis, the Kaplan–Meier survival curve with the percentage of patients free from conversion to TKA, UKA, or HTO is shown in Fig. 2. During the follow-up period, six patients in the meniscectomy group and four patients in the conservative group underwent conversion due to OA progression. The overall Kaplan-Meier probability of survival after arthroscopic meniscectomy was 99% at 5 years, 87% at 10 years, whereas that for conservative treatment was 98% at 5 years, 88% at 10 years. (*p* = 0.8). (Fig. 2) The TKA, UKA, or HTO conversion hazard was 116% Higher for the conservative group compared with the meniscectomy group but there was no statistically significant difference (*p* = 0.82).

## Discussion

This retrospective study compared two treatments, arthrosocpic meniscectomy and conservative treatment, for degenerative MMPRT. Although overall improvement was observed in the clinical results of both groups without inter-group differences, arthroscopic meniscectomy resulted in increased progression of OA in the medial compartment; however, there was no difference in the survival rate after mid-term follow-up. Thus, arthroscopic meniscectomy has no benefit compared to conservative treatment of degenerative MMPRT. The treatment options for MMPRT include conservative treatment, meniscectomy, and surgical repair. Traditionally, patients with MMPRT undergo conservative treatment or meniscectomy [26]. Meniscectomy can provide symptomatic relief, but in most cases, progression to degenerative OA does occur [3, 5]. Consequently, there has been a recent shift toward meniscal preservation along with surgical repair [4]. It is well known that acute traumatic MMPRT without OA should be repaired whenever possible to restore meniscal hoop tension and to prevent early arthritic progression [8–11]. Unfortunately, a large proportion of MMPRT cases seen in clinical practice involve degenerative tears in middle-aged or older patients [11, 12]. Hence, surgical repair is not always feasible in the population at risk of these tears [10, 13] due to substantial degeneration of the meniscal tissue and concurrent OA [2, 3, 14]. Although the overall outcomes of surgical repair have been good in some studies [13, 27, 28], Bin et al. reported that partial meniscectomy can be a good option for selected patients with good prognostic factors and for patients who are not eligible for surgical repair because of the poor quality of their meniscal tissue [1, 28]. In our study, pain and functional outcomes at first visit were significantly worse in the meniscectomy group than in the conservative group. This indicates that the greater the pain intensity, the higher the likelihood of patients choosing surgical treatment over conservative treatment. However, both meniscectomy and conservative treatment resulted in significant improvements in pain and function scores as per the VAS and IKDC scores, respectively, with no inter-group differences after an average follow-up of 6.3 years. The lack of significant differences may be due to the improvement in symptoms, including mechanical pain, with time, regardless of the treatment modality. The lack of differences in clinical outcomes despite greater progression of OA in the meniscectomy group than in the conservative group might be because the follow-up duration was not enough to detect differences in clinical outcomes. In addition, the mean value was lower in the meniscectomy group, and therefore, differences in outcome scores between treatment groups could be clinically significant, although not statistically significant. This may be a result of the analysis being underpowered.

The most important finding of this study was that OA progression was more severe in the meniscectomy group than in the conservative group (*p* = 0.03). Similar to our study, Krych et al. reported that partial meniscectomy for degenerative MMPRT provides no benefit over conservative treatment in terms of halting arthritic progression [18, 29]. Similarly, early OA development is more likely to occur after meniscectomy than after non-operative treatment [30, 31]. Meniscectomy may increase the pressure on the residual meniscus, which may worsen any subsequent articular degeneration [5, 32]. In a study by Han et al. [5], after meniscectomy for MMPRT, progression of OA on radiological examination was noted in 35% of the patients at 5–6 years after surgery. Krych et al. [18] found that 54% of partial meniscectomy patients and 34.6% of non-operative patients showed conversion to TKA at a mean of 54.3 and 30.2 months, respectively. Contrary to other studies, our study showed that the survival rate was 99% at 5 years and 87% at 10 years after meniscectomy and 98% at 5 years and 88% at 10 years after conservative treatment, possibly because meniscectomy was performed only in patients without significant malalignment or osteoarthritic change. This study has several limitations. First, it was a retrospective investigation of a small, nonrandomized case series; thus, a selection bias may be present. Moreover, the baseline pain and functional scores were low in the meniscectomy group because patients chose the treatment modality based on their symptoms and treatment characteristics. To overcome this, this study compared the degree of improvement from the baseline level to the final follow-up. Second, the follow-up period was not long enough to detect differences in the survival rate. Third, during the follow-up period, it was not clearly indicated whether another conservative treatment that could affect the clinical outcome were performed after the completion of acute treatment. Fourth, the high proportion of female patients in our study. Although MMPRT is prevalent in middle-aged female patients, sex and age can affect individual activity. This reduces the extent to which our results can be generalized. Despite these limitations, we tried to only include patients with degenerative MMPRT without significant malalignment and advanced OA to reduce selection bias to ensure objective evaluation of the effectiveness of meniscectomy for MMPRT.

## Conclusion

This study demonstrated that both conservative treatment and meniscectomy provided symptomatic relief to patients with degenerative MMPRT without advanced OA and malalignment. However, OA progression was more severe in the meniscectomy group than in the conservative group, despite the similarity in their survival rates. In light of our findings, we concluded that arthroscopic meniscectomy has no benefit compared to conservative treatment in middle-aged patients with degenerative MMPRT.

## Data Availability

The original reports, imaging studies and outpatient clinic records are retained as per normal procedure within the medical records of our institution. If someone wants to request the data from this study, please contact to namhunleeos@gmail.com.

## Abbreviations

MMPRT: Medial meniscus posterior root tear
VAS: Visual Analog Scale
IKDC: International Knee Documentation Committee
K-L: Kellgren-Lawrence
HKAA: Hip-knee-ankle angle
MPTA: Medial proximal tibial angle
TPSA: Tibial posterior slope angle
OA: Osteoarthritis
UKA: Unicompartmental knee arthroplasty
HTO: High tibial osteotomy
